# Identification of novel androgen-responsive genes by sequencing of LongSAGE libraries

**DOI:** 10.1186/1471-2164-10-476

**Published:** 2009-10-15

**Authors:** Tammy L Romanuik, Gang Wang, Robert A Holt, Steven JM Jones, Marco A Marra, Marianne D Sadar

**Affiliations:** 1 Genome Sciences Centre, British Columbia Cancer Agency, Vancouver, British Columbia, Canada

## Abstract

**Background:**

The development and maintenance of the prostate is dependent on androgens and the androgen receptor. The androgen pathway continues to be important in prostate cancer. Here, we evaluated the transcriptome of prostate cancer cells in response to androgen using long serial analysis of gene expression (LongSAGE) libraries.

**Results:**

There were 131 tags (87 genes) that displayed statistically significant (p ≤ 0.001) differences in expression in response to androgen. Many of the genes identified by LongSAGE (35/87) have not been previously reported to change expression in the direction or sense observed. In regulatory regions of the promoter and/or enhancer regions of some of these genes there are confirmed or potential androgen response elements (AREs). The expression trends of 24 novel genes were validated using quantitative real time-polymerase chain reaction (qRT-PCR). These genes were: *ARL6IP5, BLVRB, C19orf48, C1orf122, C6orf66, CAMK2N1, CCNI, DERA, ERRFI1, GLUL, GOLPH3, HM13, HSP90B1, MANEA, NANS, NIPSNAP3A, SLC41A1, SOD1, SVIP, TAOK3, TCP1, TMEM66, USP33*, and *VTA1*. The physiological relevance of these expression trends was evaluated *in vivo *using the LNCaP Hollow Fibre model. Novel androgen-responsive genes identified here participate in protein synthesis and trafficking, response to oxidative stress, transcription, proliferation, apoptosis, and differentiation.

**Conclusion:**

These processes may represent the molecular mechanisms of androgen-dependency of the prostate. Genes that participate in these pathways may be targets for therapies or biomarkers of prostate cancer.

## Background

Androgens mediate their effect through the androgen receptor (AR) and together they play integral roles in the development and maintenance of the prostate. In the absence of a functional androgen-axis during development, the prostate will fail to form[1]. The size of the prostate increases with the elevation of levels of androgens in males during puberty[2]. Androgens promote proliferation, differentiation, and survival of prostate cells[1]. Men that have used excess androgens in the form of anabolic steroids have a higher incidence of prostate cancer [3-5]. Association of prostate cancer with levels of androgens has also been reported in rodents[6,7]. Reduction of androgen in humans or dogs before puberty by castration is associated with decreased incidence of prostate cancer[8,9]. Castration of adult males causes apoptosis of prostatic epithelium, involution and reduction of the prostate [10-12]. Thus the prostate gland is an androgen-dependent organ where androgens are the predominant mitogenic stimulus[13]. The dependency of the prostate epithelium on androgens provides the underlying rationale for treating prostate cancer with chemical or surgical castration (androgen-deprivation)[14].

The AR is a ligand-activated transcription factor[15] that regulates transcription of genes that contain androgen response elements (AREs) in the upstream or downstream regulatory regions of the promoter and/or enhancer. Kallikrein 3 (*KLK3*) is an example of a gene that contains numerous functional AREs that the AR interacts with to increase transcription in response to androgens [16-19]. KLK3, also known as prostate-specific antigen (PSA), is the main tumor marker for prostate cancer and has been used clinically for 15 years[20]. Serum levels of PSA correlate with tumor volume[21]. However, as a screening and monitoring tool for prostate cancer, serum PSA levels are subject to false positives and false negatives[20].

Identification of the genes that change in expression in response to androgen in prostate cells is essential for the understanding of androgen-dependency of the normal prostate and the proliferation, survival, and hormonal progression of prostate cancer. There are several studies that have investigated genes that alter expression in response to a changing androgen-axis using SAGE [22-24]. Here we highlight several key differences in the current experimental design from previous studies: 1) a physiological concentration of metabolically stable androgen (R1881) was employed *in vitro*; 2) the transcriptome was catalogued using LongSAGE[25] opposed to (short)SAGE[26] because it generates lengthier tags allowing increased confidence in tag-to-gene mapping, and leaves fewer tags unmapped[25]; 3) the transcriptome of human prostate cancer cells was examined instead of murine cells [22]; 4) sequencing depth was increased by approximately 1.5-2 times more tags relative to other studies [23,24] to improve the potential for novel findings; 5) *transcript *expression was validated using an alternative assay as opposed to protein expression [24], and tens of novel genes were validated as opposed to only two[23]. Thus, we apply LongSAGE for the first time to create transcript libraries of prostate cancer cells maintained in the presence or absence of androgen. These libraries are publicly available at Gene Expression Omnibus. We describe 24 genes never before identified or validated to alter expression in response to androgen treatment. These genes were: *ARL6IP5, BLVRB, C19orf48, C1orf122, C6orf66, CAMK2N1, CCNI, DERA, ERRFI1, GLUL, GOLPH3, HM13, HSP90B1, MANEA, NANS, NIPSNAP3A, SLC41A1, SOD1, SVIP, TAOK3, TCP1, TMEM66, USP33*, and *VTA1*. Statistically significant changes in expression of *ARL6IP5, CAMK2N1, ERRFI1, HSP90B1*, and *TAOK3 *in response to reduced levels of circulating androgens were measured using *in vivo *samples.

## Results and discussion

### Summary of LongSAGE libraries

LongSAGE was employed to obtain quantitative gene expression profiles of human prostate cancer cells treated with or without synthetic androgen R1881. LNCaP human prostate cancer cells were chosen as the model cell line for evaluating androgen signaling because they respond to androgens, express a functional although mutated (T877A) AR, they can be grown *in vitro *as a monolayer or *in vivo *as a xenograft or in the Hollow Fiber model [27-29]. LNCaP cells have been used extensively in prostate cancer research. The time of 16 hours for treatment and concentration of R1881 (10 nM) were chosen based upon optimal induction of levels of *KLK3 *mRNA [30].

LongSAGE libraries were sequenced to a total of 121,760 (R1881) and 103,391 (vehicle) tags (Table 1). The libraries were filtered on several levels to leave only useful tags for analysis. First, bad tags were removed if they contained at least one N-base call in the LongSAGE tag sequence. Notably, when bad tags were filtered the percentages of duplicate ditags in the R1881 and vehicle LongSAGE libraries were 6% and 5%, respectively. Early SAGE studies suggest duplicate ditags likely represent polymerase chain reaction (PCR) artifacts due to the low probability the same two tags will ligate together to form ditags[26]. However, with LongSAGE library sequencing and highly expressed transcripts, this probability becomes significant[31]. A recent study[32] suggests that discarding duplicate ditags in LongSAGE analysis may introduce a bias affecting the fold differences in tag expression between libraries for all tags observed at a frequency >(113-224)/100,000. Therefore, we opted to retain duplicate ditags. PHRED software was used to call bases for the sequencing of the LongSAGE tags[33,34]. PHRED has a small, but significant error rate in base-calls. To ascertain which tags potentially contained these erroneous base-calls, we calculated a tag sequence quality factor (QF) and probability[35]. The second line of filtering removed LongSAGE tags with probabilities less than 0.95 (QF < 95%). Linkers of known sequence were introduced into SAGE libraries as primers for amplifying ditags prior to concatenation[26]. These linker sequences were designed so they do not map to the human genome. At a low frequency, linkers ligate to themselves creating linker-derived tags (LDTs). These LDTs do not represent transcripts and are removed from the LongSAGE libraries. After filtering, there were 97,981 total useful tags representing 23,828 tag sequences in the R1881 LongSAGE library, and 85,861 total useful tags representing 24,592 tag sequences in the vehicle LongSAGE library. Due to redundancy in the expressed sequences, the combined number of useful tag types in the R1881 and vehicle LongSAGE libraries was 38,574. The remainder of the data analysis in this manuscript was carried out using this filtered data.

**Table 1:** Composition of LongSAGE libraries

**Unfiltered**	**Library**	**R1881**	**Vehicle**
	Unfiltered Total Tags	121,760	103,391
	No. of Bad Tags	528	383

Minus Bad Tags	Total Tags	121,232	103,008
	Tag Types	33,385	31,764
	No. of Duplicate Ditags	6,763	5,193
	% of Duplicate Ditags	5.579	5.041
	Average QF^*r *^of Tags	8 9.64	89.67
	No. of Tags QF<95%	22,816	17,095

QF ≥ 95%	Total Tags	98,416	85,913
	Tag Types	23,830	24,594
	Total Tags Combined	184,329	
	Tag Types Combined	38,576	
	No. of LDTs^*s *^Type I	219	34
	No. of LDTs Type II	216	18

Minus LDTs	Total Tags	97,981	85,861
	Tag Types	23,828	24,592
	Total Tags Combined	183,842	
	Tag Types Combined	38,574	

*r *QF, Quality Factor

*s *LDTs, Linker-derived Tags

### Tag frequency and transcript abundance

Tag frequency spanned over three orders of magnitude corresponding to transcript abundance of 5 to 8,746 copies per cell (based on minimum and maximum observed tag counts of 1 and 1714; see Table 2 legend)[36]. The distribution of LongSAGE tag frequencies per 100,000 tags revealed the majority (64 and 67%) of tag types in each LongSAGE library (R1881 and vehicle, respectively) were singletons (tags counted only once). This result was consistent with other published SAGE libraries reporting 66% singletons[37]. Singletons can represent very low abundance transcripts (≤ 5 transcript copies per cell) or PCR/sequencing errors. Estimates indicate that less than 17% of LongSAGE tags in a library contain PCR/sequencing errors[38]. Coincidently, 17% of the total tags in the R1881 and vehicle LongSAGE libraries roughly equal the number of singletons in each LongSAGE library (Table 2). Although initial estimates suggest 6.8-10% of shortSAGE tags contain PCR/sequencing errors, more recent experimental evidence suggests the actual error rate is much lower (≤ 2%)[39]. This implies that an error rate of 17% may also be an overestimate for LongSAGE tags. Tag types counted 2-4 times per 100,000 tags (10-20 transcript copies per cell) and 5-9 times per 100,000 tags (25-45 transcript copies per cell) were the second and third most common groups of tag types, respectively. Generally, high frequency tags were less common. The majority of total tags in each LongSAGE library were derived from a few tag types detected between 10-99 times per 100,000 tags (50-495 transcript copies per cell).

**Table 2:** Characteristics of LongSAGE tag frequency distribution

Tag Frequency & Abundance	Tag Count per 100,000^*t*^	≤1	2-4	5-9	10-99	100-999	≥1,000
	Transcript Copies per Cell^*u*^	≤5	10-20	25-45	50-495	500-4,995	≥5,000
	% Transcript Abundance in Cell^*v*^	≤0.001	0.002-0.004	0.005-0.009	0.01-0.099	0.1-0.999	≥1
							
R1881	Total Tags	15,141	13,985	11,055	32,800	21,971	3,029
	Tag Types	15,141	5,464	1,703	1,417	101	2
							
Vehicle	Total Tags	16,562	1 0,229	11,633	26,466	18,453	2,518
	Tag Types	16,562	4,427	2,195	1,313	93	2
% of Tags that Map as Transcription Factors^*α*, *z*, *δ*^		9.14	8.94	7.95	6.0	0	0
	% of Tags that Map^*χ*, *β*, *δ*^	29.40	57.82	76.22	83.1	85	100^γ^
% of Tags Significantly Differentially Expressed^*ε*, *a*, *δ*^		0.4	1.15	16.17	25.38	58.12	100

*t *Tag count per 100,000 = (observed tag count/total tags in the library) × 100,000

*u *Transcript copies per cell^*w *^= (observed tag count/total tags in the library) × 500,000

*v *% Transcript abundance in cell^*w *^= (transcript copies per cell/500,000) × 100%

*w *Calculation based on ~500,000 transcripts in a cell [36]

α % of tags that map as transcription factors = (no. of genes with "transcription regulation acivity"/no. of genes with unambiguous sense mappings and GO terms) × 100%

^*z*^Mapped unambigously sense toRefSeqand subjected to Gene Ontology (GO) analysis

δ Tag types from each tag frequency class of R1881 and vehicle LongSAGE libraries were combined

χ % of tags that map = (no. of genes with sense mappings/combined total tag types) × 1 00%

β Mapped sense (incl. ambiguous) to RefSeq

γ One tag was mapped sense using Ensembl gene

ε % of tags significantly differentially expressed = (no. of significantly differently expressed tag types in class/combined total tag types in class) × 100%

*a *Statistics according to the Audic and Claverie test statistic (p ≤ 0.001)

### Mapping distribution of LongSAGE tags

When mapped tags (v38 Ensembl) were clustered to amalgamate 1-off tags (see Methods, Gene Expression Analysis for a description) and tags that mapped ambiguously were removed, the tag types in the R1881 and vehicle LongSAGE libraries represented 7,484 genes and 7,441 genes, respectively (Table 3). Tag types that mapped ambiguously constituted 13% (R1881 and vehicle), while 36% (R1881) and 35% (vehicle) of tag types did not map to the genome (Table 3). Due to the fact that these tags were clustered, the majority of the tags that did not map to the genome probably represent true unannotated transcripts rather than PCR/sequencing errors. Approximately 28% of tags in each LongSAGE library mapped to the opposite strand of known genes. These LongSAGE tags either represent transcription from previously undescribed coding regions or true antisense transcripts. Each LongSAGE library contained tags representing transcripts from 32% of the genes in the Ensembl gene database. This percentage is indicative of the depth of coverage of the transcriptome achieved with LongSAGE. Alternatively, this percentage indicates that one third of known Ensembl genes were expressed in LNCaP cells under these experimental conditions. This percentage is substantial when considering tag types from the Mouse Atlas Project (8.55 million total LongSAGE tags generated from 72 libraries of mouse development) mapped to 57% of the Ensembl transcript database[35]. Approximately 63% (R1881) and 61% (vehicle) of the genes that mapped to Ensembl's database were associated with more than one tag type to suggest that most gene expression was represented by transcript variants which is consistent with previous observations[35]. When the mapped LongSAGE tags (Reference Sequence, RefSeq; May 18, 2006) were clustered to amalgamate 1-off tags and tags that mapped ambiguously were removed, 53% of tags mapped solely to known exons, 9% solely to known introns (novel transcript variants), and 38% to intergenic regions (novel genes or transcript variants).

**Table 3:** LongSAGE tag mappings^*x*^

**Library**	**No. of Tag Types that Mapped Unambiguously to (Genes)**	**No. of Tag Types that Mapped Ambiguously**	**No. of Tag Types that Did Not Map**	**Total No. of Tag Types (Clustered)^*y*^**
R1881	14,587 (7,484)	3,754	10,215	28,556
Vehicle	13,626 (7,441)	3,286	9,066	25,978

*x *Ensembl gene (v38) was used for mapping

*y *Clustering amalgamated 1-off tags with likely 'parent' tags to improve the mapping capability of LongSAGE tags Clustering altered the number of tag types without changing the total number of tags in the libraries

The two most abundant tag types in the LongSAGE libraries were shared by both libraries. The first highly abundant LongSAGE tag mapped to human mitochondrial NADH ubiquinone oxidoreductase chain 4. This gene is also highly expressed in other human tissues (i.e., cardiac tissue; SAGE Genie, ). The protein product of this gene transfers electrons from NADH to ubiquinone to generate adenosine triphosphate as metabolic energy. Using the Ensembl database, the second most abundant LongSAGE tag mapped to a non-coding gene of human mitochondria. In contrast to the higher abundance classes, the lower abundance classes were enriched for LongSAGE tags that mapped to genes with functions in regulating transcription (Table 2). This is particularly significant because the percentages of LongSAGE tags that mapped to the genome in the lower abundance class were reduced compared to the higher abundance classes (Table 2). Together this implies that the number of tags that map to genes with a function in transcription may be underestimated, as low abundance tags may be underrepresented.

### Differential gene expression

**Figure 1:**
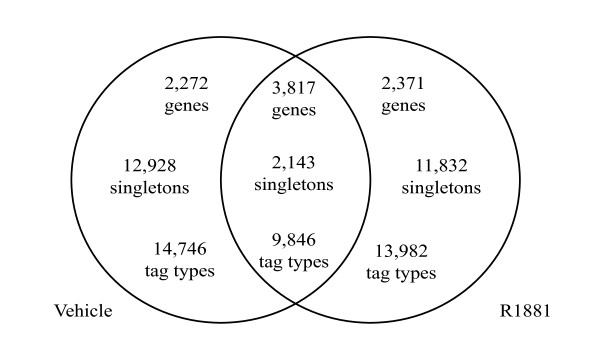
**Relationship between LongSAGE library compositions**. The Venn Diagram shows the tag types and genes exclusive to, and shared by each LongSAGE library, R1881 and vehicle. Tags were mapped unambiguously sense to RefSeq transcripts and redundant mappings were removed. Singletons are tags counted only once in each library, but may be common to both libraries.

Venn analysis identified that 36% and 38% of tag types were exclusive to the R1881 or vehicle LongSAGE libraries, respectively (Figure 1). The unique expression of tag types indicates differential expression depending upon androgen stimulation. The biological relevance of this differential expression is complicated by the fact that 85% (R1881) and 88% (vehicle) of these exclusive LongSAGE tags were singletons. Consistent with our observation that low abundance tags did not map as readily to the genome, the mutually exclusive tags also did not map as readily as tags shared between both libraries. Only 17% and 15% of tags exclusive to R1881 and vehicle LongSAGE libraries, respectively, mapped unambiguously sense to RefSeq, in contrast to 39% of shared tags. We therefore, concentrated on genes for which the tag abundance allowed for the determination of statistically significant changes in transcript abundance.

**Figure 2:**
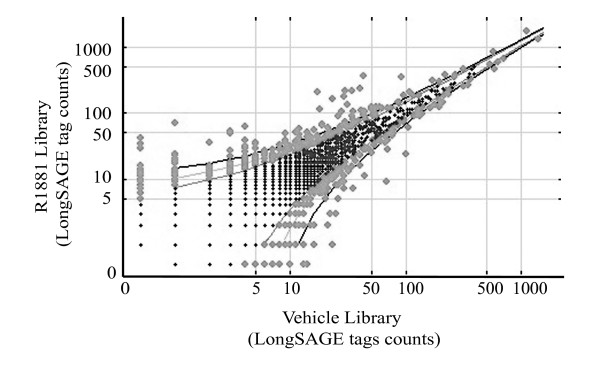
**Confidence intervals highlight expressed tag types with non-linear relationships between LongSAGE libraries**. Scatter plot dots represent tag types and their placement on the axis indicates the frequency of observation in either of the LongSAGE libraries. Tag types that fall outside the confidence interval (CI) lines are considered statistically significantly differentially expressed (Audic and Claverie test statistic); outer line, 99.9% CI; middle line, 99% CI; and inner line, 95% CI.

A scatter plot illustrates observed tag counts in LongSAGE libraries relative to the confidence intervals (CIs; 95%, 99%, and 99.9%) of respective p-values (p ≤ 0.05, 0.01, and 0.001) by Audic and Claverie statistics[40] (Figure 2). 891 tags were differentially expressed (p ≤ 0.05) between the two LongSAGE libraries (Figure 2 and Table 4). LongSAGE tags statistically (p ≤ 0.001) differentially represented between the libraries were enriched in the higher abundance classes compared to the lower abundance classes (Table 2). Additionally, 90% of the LongSAGE tags were statistically (p ≤ 0.001) differentially represented between the libraries with ≥ 2-fold differences, compared to only 17% of tags with p-values greater than 0.001 (p > 0.001).

**Table 4:** Number of tag types found to be significantly differentially expressed between R1881 and vehicle libraries^*a*^

**Direction of Change**	**p ≤ 0.001**	**p ≤ 0.01**	**p ≤ 0.05**
Up Regulated	83	196	455
Down Regulated	48	120	436
			
Total	131	316	891
% of All Tag Types	0.34%	0.82%	2.31%

*a *Statistics according to the Audic and Claverie test statistic

A stringent p-value cutoff (p ≤ 0.001), not corrected for multiple tests, was employed prior to validation of changes in expression of a gene in response to androgen. LongSAGE tags that were differentially expressed, but mapped ambiguously to more than one gene, and/or differed by less than 2-fold between the treatment groups, were excluded from analysis. Application of these criteria reduced the LongSAGE tags from 131 to 93. These 93 tags represented 87 genes. Analysis of differentially expressed LongSAGE tags revealed that 54 LongSAGE tags that mapped to 52 genes were previously known to change in expression in the direction observed in response to androgen in prostate cancer cells. Of these, the expression of 41 genes increased as expected, including the well-known androgen-regulated gene, *KLK3 *(Table 5). The expression of 11 genes decreased in response to androgen and were consistent with previous reports (Table 6). Genes previously not reported to alter expression in response to androgen in prostate cancer cells were represented by 39 LongSAGE tags. These tags represented the expression of 20 genes that were increased, excluding mappings to non-coding and intergenic regions, (Table 7), and expression of 15 genes that was decreased (Table 8) in response to androgen. The 93 tags were represented by 87 genes because one tag did not map to the human genome (Table 7) and two tags mapped to intergenic regions of the human mitochondrial genome (Tables 7 and 8). Three genes were represented twice in the tables (*CAMK2N1*, *PPAP2A*, and *SORD*). One gene, *KRT8*, was categorized in both the known and not previously known categories due to the sense of the mapping (Tables 5 and 8).

**Table 5:** LongSAGE tags corresponding to genes known to increase expression in response to androgen in LNCaP cells^*a*, *n*^

	**Tags/100,000^*d*, *t*^**					
					
**LongSAGE Tag Sequence**	**Vehicle**	**R1881**	**Fold^*c*, *d *^Change**	**RefSeq/Ensembl Access. No.**	**HGNC Gene Symbol**	**Description^φ^**
GTGACAAGTGACAGAGT	1	19	20	NM_007011	*ABHD2*	Abhydrolase domain containing 2, transcript variant 1
ACGTCACCATTTTTAAC	1	24	20	NM_004457	*ACSL3*	Acyl-CoA synthetase long-chain family member 3, transcript variant 1
TACTTTATAAGTATTGG	14	59	4.2	NM_006988	ADAMTS1*	ADAM metallopeptidase with thrombospondin type 1 motif, 1
TAGCTCTATGGGGGGAG	35	75	2.1	NM_000701	*ATP1A1*	ATPase, Na+/K+ transporting, alpha 1 polypeptide, transcript variant 1
GTTGTGGTTAATCTGGT	48	109	2.3	NM_004048	*B2M*	Beta-2-microglobulin
ACTTAAGGAACTTATCT	14	42	3.0	NM_015415	*BRP44*	Brain protein 44
AAAGGAAAATAAAAATT	3	27	9	NM_018455	CENPN*	Centromeric protein N
CTGTGATGTGACTCCTG	5	30	6	NM_030806	*Clorf21*	Chromosome 1 open reading frame 21
CAGATGAGATGTGAGCT	5	33	7	NM_130898	CREB3L4*	cAMP responsive element binding protein 3-like-4
TGTTTATCCTAAACTGA	21	115	5.5	NM_020548	*DEI*	Diazepam binding inhibitor (GAB A receptor modulator, acyl-Coenzyme A binding protein)
TCCCCGTGGCTGTGGGG	106	356	3.36	NM_014762	*DHCR24*	24-dehydrocholesterol reductase
GAAATTAGGGAAGCCTT	9	34	4	NM_015036	*ENDOD1*	Endonuclease domain containing 1
AGATCCTACTTAGTATG	16	51	3.2	NM_004462	*FDFT1*	Farnesyl-diphosphate farnesyltransferase 1
GTTCCAGTGAGGCCAAG	3	50	20	NM_004117	FKBP5*	FK506 binding protein 5
ACCTAGCCACTGCTGGG	1	24	20	NM_002247	*KCNMA1*	Potassium large conductance calcium-activated channel, subfamily M, alpha member 1, transcript variant 2
GGATGGGGATGAAGTAA	50	366	7.3	NM_001648	KLK3*	Kallikrein 3, (prostate-specific antigen), transcript variant 1
CCTCCAGCTACAAAACA	35	223	6.4	NM_002273	*KRT8*	Keratin 8
TAAAATATTGAAGTGTC	ND^*b*^	42	40	NM_015541	LRIG1*	Leucine-rich repeats and immunoglobulin-like domains 1
TCCCTGAGCACCATTGC	ND	35	40	NM_015261	NCAPD3*	Non-SMC condensin complex subunit D3
GGACTTTCCTTCCCTCT	1	72	70	NM_006096	*NDRG1*	N-myc downstream regulated gene 1
TTTAGGTAAACGAAAGC	19	56	2.9	NM_014445	N/A^*q*^	Stress-associated ER protein 1
AGGTTTTGCCTCATTCC	13	38	2.9	ENSG00000196930	N/A^*q*^	Similar to Vesicle-associated membrane protein-associated protein A mRNA
ATGCAGCCATATGGAAG	20	208	10	NM_002539	*ODC1*	Ornithine decarboxylase 1
GCCAAGGGGCCAGCTGC	17	45	2.6	NM_002541	*OGDH*	Oxoglutarate (alpha-ketoglutarate) dehydrogenase (lipoamide), nuclear gene encoding mitochondrial protein, transcript variant 1
TAATTTTTACTTTGTAC	5	39	8	NM_017906	PAK1IP1*	PAK1 interacting protein 1
TATGTAATATGCTTTCT	27	164	6.1	NM_003711	*PPAP2A*	Phosphatidic acid phosphatase type 2A, transcript variant 1
AAACACCAACAACTGGG	5	31	6	NM_003711	*PPAP2A*	Phosphatidic acid phosphatase type 2A isoforms 1 and 2
GTGTTTACGTGATCCAC	1	18	20	NM_004578	*RAB4A*	RAB4A, member RAS oncogene family
TATGTATAAATGGACCT	ND	16	20	NM_021205	RHOU*	Ras homolog gene family, member U
TTTGAAATGAGGTCTGT	14	48	3.4	NM_002970	*SAT*	Spermidine/spermine N1-acetyltransferase
GCAACAGCAATAGGATT	3	22	7	NM_014302	*SEC61G*	Sec61 gamma subunit, transcript variant 1
GCGCTGGAGTGAGATGG	59	126	2.1	NM_031287	*SF3B5*	Splicing factor 3B, subunit 5, l0kDa
GGATTTGAACATATGAA	ND	13	10	NM_033102	*SLC45A3*	Solute carrier family 45, member 3
ACCTTGTGCCCGATTCT	47	238	5.1	NM_003104	*SORD*	Sorbitol dehydrogenase
AAAATCTGCCACTCAGG	ND	1 2	10	NM_003104	*SORD*	Sorbitol dehydrogenase
GTGCAGGGAGACATCTG	3	55	20	NM_012391	*SPDEF*	SAM pointed domain containing ets transcription factor
TTAAGGGATGATGGCTT	ND	1 2	10	NM_024636	*STEAP4*	STEAP family member 4
TACTACAGCTATATTTG	1 6	52	3. 3	NM_016192	*TMEFF2*	Transmembrane protein with EGF-like and 2 follistatin-like domains 2
TGATGTCTGGTCTGAAT	1	1 7	20	NM_020182	*TMEPAI*	Transmembrane, prostate androgen induced RNA, transcript variant 1
CAAATAAATTATGCGAT	5	64	10	NM_005656	*TMPRSS2*	Transmembrane protease, serine 2
TGAAAAGCTTAATAAAT	7	28	4	NM_005079	*TPD52*	Tumor protein D52, transcript variant 3
TTAAAGATTTAGACACC	10	36	3. 6	ENSG00000140416	*TPM1*	Tropomyosin 1 apha chain
TTCTCTACACAATTGTA	6	36	6	NM_006022	*TSC22D1*	TSC22 domain family, member 1, transcript variant 1

*a *Statistics according to the Audic and Claverie test statistic (p ≤ 0.001)

*b *ND, not detected

*c *ND tags were assigned a value of 1 when calculating fold

*d *Appropriate significant figures are displayed

*n *Ambiguously mapped tags and tags with a fold change less than 2-fold have been excluded from the table

*q *N/A = there is no HGNC approved gene symbol for this tag

*t *Tag count per 100,000 = (observed tag count/total tags in the library) × 100,000

φ In cases where a tag mapped to >1 transcript variant of the same gene the RefSeq accession number for transcript variant 1 was displayed

* Gene further characterized in this paper

**Table 6:** LongSAGE tags corresponding to genes known to decrease expression in response to androgen in LNCaP cells^*a*, *n*^

	**Tags/100,000^*d*, *t*^**					
					
**LongSAGE Tag Sequence**	**Vehicle**	**R1881**	**Fold Change^*c*, *d*, *j*^**	**RefSeq/Ensembl Access. No.**	**HGNC Gene Symbol**	**Description^φ^**
CAAAAGCTTATTCTTGT	29	3	-10	NM_016613	*C4orf18*	Chromosome 4 open reading frame 18, transcript variant 2
TCACACAGTGCCTGTCG	19	1	-20	NM_020311	CXCR7*	Chemokine orphan receptor 1
ACAAACCCCCACCCCAG	41	7	-6	NM_013330	*NME7*	Non-metastatic cells 7, protein expressed in, transcript variant 1, Nucleoside diphosphate kinase
AATCTCTCAATTATAGG	34	9	-4	NM_006183	NTS*	Neurotensin
ATCAACTGGAGGCTCAG	15	ND^*b*^	-20	NM_005013	*NUCB2*	Nucleobindin 2
CCAAAATTAGGAAAAAC	15	1	-20	NM_002577	*PAK2*	p21 (CDKNlA)-activated kinase 2^*k*^
TTACGTTTGGGAAAAAT	19	2	-9	NM_032971	*PCDH11Y*	Protocadherin 11 Y-linked, transcript variant a^*k*^
TGACTTTGGTGCCGTTA	12	ND	-10	NM_003629	*PIK3R3*	Phosphoinositide-3-kinase, regulatory subunit 3 (p55, gamma)
AGCAAATATGTCAAGGG	47	16	-2.9	NM_182948	PRKACB*	Protein kinase, cAMP-dependent, catalytic, beta, transcript variant 1
GACTATTCCATATTAAA	27	1	-30	NM_018412	ST7*	Suppression of tumorigenicity 7, transcript variant A
GAGGGTTTTAAATGGAG	79	9	-9	NM_001077	*UGT2B17*	UDP glucuronosyltransferase 2 family, polypeptide B17

*a *Statistics according to the Audic and Claverie test statistic (p ≤ 0.001)

*b *ND, not detected

*c *ND tags were assigned a value of 1 when calculating fold change

*d *Appropriate significant figures are displayed

*j *Negative fold change value indicates down-regulation in response to R1881

*k *Tag has a single base pair permutation, insertion, or deletion with respect to gene

*n *Ambiguously mapped tags and tags with a fold change less than 2-fold have been excluded from table

*t *Tag count per 100,000 = (observed tag count/total tags in the library) × 100,000

φ In cases where a tag mapped to >1 transcript variant of the same gene the RefSeq accession number for transcript variant 1 was displayed

* Gene further characterized in this paper

**Table 7:** LongSAGE tags corresponding to genes not previously reported to increase expression in response to androgen in LNCaP cells^*a*, *n*^

	**Tags/100,000^*d*, *t*^**					
					
**LongSAGE Tag Sequence**	**Vehicle**	**R1881**	**Fold Change^*c*, *d*^**	**RefSeq/Ensembl Access. No.**	**HGNC Gene Symbol**	**Description^φ^**
TCTTTATTAGAAAAAAA	ND^*b*^	16	20	NM_014265	*ADAM28*	ADAM metallopeptidase domain 28, transcript variant 1^*k*^
AGGAGCAAAGGAAGGGG	51	107	2.1	NM_000713	BLVRB*	Biliverdin reductase B (flavin reductase (NADPH))
TTTTGGGGGCTTTTAGC	16	44	2.8	NM_198446	Clorf122*	Chromosome 1 open reading frame 122
GGGCCCCAAAGCACTGC	22	69	3.1	NM_199249	C19orf48*	Chromosome 19 open reading frame 48
CCCCAGTTGCTGATCTC	24	60	2.5	NM_001003962	CAPNS1*	Calpain, small subunit 1, transcript variant 2
CTTAAGAAAAATGCACT	1	23	20	NM_018948	ERRFI1 *	ERBB receptor feedback inhibitor 1
TACAGTATGTTCAAAGT	13	52	4.0	NM_002065	GLUL*	Glutamate-ammonia ligase (glutamine synthetase), transcript variant 1^*g*, *i*^
TTAATAGTGGGGCTTTC	10	39	3.9	NM_022130	GOLPH3*	Golgi phosphoprotein 3 (coat protein)
GCCAGGGCGGGCCACTG	ND	16	20	NM_178580	HM13*	Histocompatibility (minor) 13, transcript variant 2
GAGGAAGAAGAAGCAGC	ND	14	10	NM_003299	HSP90B1*	Heat shock protein 90kDa beta (Grp94), member 1
GGCAAGGGGGGTCCCCA	1	20	20	NM_002273	*KRT8*	Keratin 8^*m*^
ACTCCAAAAAAAAAAAA	41	81	2.0	XM_376154	N/A^*q*^	Similar to 40S ribosomal protein S15 (RIG protein), transcript variant 1
GGGTTGGCTTGAAACCA	6	30	5	ENSG00000210151	N/A	Non-coding predicted mitochondrial gene ^*m*^
GAGAGCTCCCGTGAGTG	72	122	1.7	NC_001807^*P*^	N/A	Intergenic region of mitochondrial genome
TCGGACGTACATCGTTA	40	223	5.6	No map	N/A	N/A
GCAAAAAAATCAAGTCT	22	66	3.0	NM_018946	NANS*	N-acetylneuraminic acid phosphate synthase (sialic acid synthase)
TCTTTTAGCCAATTCAG	2	36	20	NM_006167	*NKX3-1*	NK3 transcription factor related, locus 1 ^*m*^
TACTTTTGGCCTGGCTG	6	35	6	NM_173854	SLC41A1*	Solute carrier family 41, member 1
GAGAGCCTCAGAATGGG	5	26	5	NM_016281	TAOK3*	TAO kinase 3
GAAGTTATGAAGATGCT	41	106	2.6	NM_030752	TCP1*	T-complex protein 1, transcript variant 1
CAGTTCTCTGTGAAATC	40	93	2.3	NM_016127	TMEM66*	Transmembrane protein 66
ATGGCTTTGTTTTGGTT	ND	14	10	NM_201624	USP33*	Ubiquitin specific protease 33, transcript variant 2

*a *Statistics according to the Audic and Claverie test statistic (p ≤ 0.001)

*b *ND, not detected

*c *ND tags were assigned a value of 1 when calculating fold change

*d *Appropriate significant figures are displayed

*e *Gene family, but not this family member, previously described to change expression in response to androgens

*g *Protein known to change expression in reponse to androgens

*h *Gene known to change expression in response to androgens, but in the opposite direction as reported here

*i *Gene known to change expression in response to androgens in cells other than prostate

*k *Tag has a single base pair mutation, insertion, or deletion with respect to gene map

*m*Tag maps to the strand opposite of the gene

*n *Ambiguously mapped tags and tags with a fold change less than 2-fold have been excluded from the table

*p *NC_001807, refers to the complete genome of mitochondria in humans

All mitochondrial genes in the RefSeq database are assigned the same accession number by NCBI *q *N/A, there is no HGNC approved gene symbol or description for this tag *t *Tag count per 100,000 = (observed tag count/total tags in the library) × 100,000

φ In cases where a tag mapped to >1 transcript variant of the same gene the RefSeq accession number for transcript variant 1 was displayed * Gene further characterized in this paper

**Table 8:** LongSAGE tags corresponding to genes not previously reported to decrease expression in response to androgen in LNCaP cells^*a*, *n*^

	**Tags/100,000^*d*, *t*^**					
					
**LongSAGE Tag Sequence**	**Vehicle**	**R1881**	**Fold^*c*, *d*, *j *^Change**	**RefSeq/Ensembl Access. No.**	**HGNC Gene Symbol**	**Description^φ^**
GTCTAGAATCTGTACCC	29	8	-4	NM_006407	ARL6IP5*	ADP-ribosylation-like factor 6 interacting protein 5
TCAAGAGCCGAAGGAAT	12	ND^*b*^	-10	NM_014165	C6orf66*	Chromosome 6 open reading frame 66
GTATTTGCAAAAATGCC	118	24	-4.9	NM_018584	CAMK2N1*	Calcium/calmodulin-dependent protein kinase II inhibitor 1
AAAAGAGAAAGCACTTT	30	5	-6	NM_018584	CAMK2N1*	Calcium/calmodulin-dependent protein kinase II inhibitor 1
TTATAACTGAATTTAGT	51	11	-4.6	NM_006835	CCNI*	Cyclin I^*i*, *h*^
GCCAGGAGAAGGGACAG	34	7	-5	NP_775809	*CNBD1*	N/A^*m*^
TGGTACTCATTTCAGGC	12	ND	-10	NM_015954	DERA*	2-deoxyribose-5-phosphate aldolase homolog
AATCATAATGGATTCTT	16	ND	-20	NM_024641	MANEA*	Mannosidase, endo-alpha
CTAAGACTTCACCAGCC	19	2	-10	ENSG00000210082	N/A ^*q*^	Non-coding predicted mitochondrial rRN A gene^*k*^
CATTTGGTATTTTCGTC	30	8	-4	NC_001807^*P*^	N/A	Intergenic region of mitochondrial genome
GTATTTCAGTGTCTGTC	33	9	-4	NM_015469	NIPSNAP3A*	Nipsnap homolog 3A
GTGTGTGGTGCCCCCAG	23	5	-5	NM_024066	PRNPIP*	Prion protein interacting protein
GTGTTAACCAGCTAAAG	122	60	-2.0	NM_002948	*RPL15*	Ribosomal protein L15
GCACAAGAAGATTAAAA	58	25	-2.3	NR_002746	*SNORD47*	Small nucleolar RNA, C/D box 47 on chromosome 1
AAAAAGCAGATGACTTG	77	37	-2.1	NM_000454	SOD1*	Superoxide dismutase 1, soluble (amyotrophic lateral sclerosis 1 (adult))
GTTTGGTTATAAATTCT	26	3	-10	NM_148893	SVIP*	Hypothetical protein DKFZp313A2432, transcript variant 1
TATTAGAGAATGAAAAG	17	2	-9	NM_016485	WA1*	VPS20-associated 1 homologue

*a *Statistics according to the Audic and Claverie test statistic (p ≤ 0.001)

*b *ND, not detected

*c *ND tags were assigned a value of 1 when calculating fold change

*d *Appropriate significant figures are displayed

*h *Gene known to change expression in response to androgens, but in the opposite direction as reported here

*i *Gene known to change expression in response to androgens in cells other than prostate

*j *Negative fold change value indicates down-regulation by R1881

*k *Tag has a single base pair permutation, insertion, or deletion with respect to gene

*m*Tag maps to the strand opposite of the gene

*n *Ambiguously mapped tags and tags with a fold change less than 2-fold have been excluded from the table

*p *NC_001807 refers to the complete genome of mitochondria in humans

All mitochondrial genes in the RefSeq database are assigned the same accession number by NCBI *q *N/A = there is no HGNC approved gene symbol for this tag *t *Tag count per 100,000 = (observed tag count/total tags in the library) × 100,000

φ In cases where a tag mapped to >1 transcript variant of the same gene the RefSeq accession number for transcript variant 1 was displayed * Gene further characterized in this paper

Interestingly some antisense tags were identified as differentially expressed in response to androgen. Antisense to NKX3-1 is of particular note. Transcription of this gene is regulated by androgen in a time- and concentration-dependent manner [41] with an ARE confirmed in its enhancer region [42]. Anti-sense RNA is involved in transcriptional silencing of sense transcript, imprinting control, post-transcriptional down-regulation of sense transcript or even stabilizing/promotion of the expression of the sense transcript [43]. In the case of NKX3-1, antisense transcript may be a negative feedback mechanism; however, this remains to be determined.

### Validation of changes in gene expression in response to androgen

**Figure 3:**
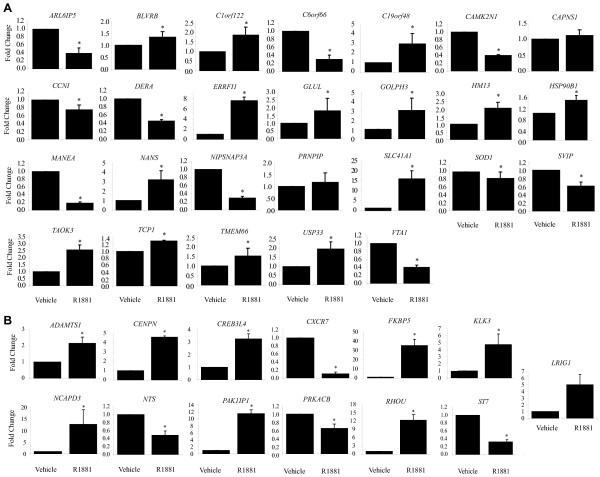
**Androgen regulation of genes as measured by qRT-PCR**. ***A ***Candidate genes not previously implicated to change expression in response to androgens in prostate cancer cells, and ***B ***Genes known to change levels of expression in response to androgens. LNCaP cells were treated for 16 hours prior to harvesting RNA, and analysing mRNA levels by qRT-PCR. Fold-change was calculated by normalizing the mean normalized expression (MNE) of transcripts in R1881-treated cells to the mock vehicle-treated cells. In doing this, the vehicle treatment fold-change became one and standard deviation (SD) zero. Error bars represent ± SD for biological sextuplets. [*] Asterisk indicates significant differential gene expression according to the Two-Sample Student's T-test (p ≤ 0.05) for unequal variance.

Quantitative real time-polymerase chain reaction (qRT-PCR) was used to validate changes in gene expression in response to androgen of 39 (13 known; 26 novel) of the 87 total genes identified by LongSAGE. Of the 35 genes previously not reported to change expression in response to androgens in prostate cancer cells, only 26 were quantified by qRT-PCR, because technical limitations and gaps in the transcriptome databases prevented the analysis of 9 genes. That is, specific qRT-PCR primers could not be designed due to repetition in the genome, or because the tag mapped to an unannotated transcript variant. There were 24 of the 26 (92%) novel genes that displayed statistically significant differential expression in response to androgen as measured by qRT-PCR (Figure 3A). *BLVRB, C19orf48, C1orf122, ERRFI1, GLUL, GOLPH3, HM13, HSP90B1, NANS, SLC41A1, TAOK3, TCP1, TMEM66*, and *USP33 *all increased levels of expression in response to androgen, while *ARL6IP5, C6orf66, CAMK2N1, CCNI, DERA, MANEA, NIPSNAP3A, SOD1, SVIP*, and *VTA1 *decreased in response to androgen (Figure 3A). Under the experimental conditions and primers used, we did not measure statistically significant changes in expression of *PRNPIP *and *CAPNS1*. A false discovery rate (FDR)[44] of 29% was expected of the LongSAGE data based on the Audic and Claverie p-value ≤ 0.001. This FDR represents the anticipated percentage of type I errors (i.e., false positives). We observed only 2/26 (8%) false positives, suggesting that the other filter parameters (e.g., ≥ 2-fold difference in expression level) may have the increased the chances of validation by qRT-PCR. Moreover, the expression trends for all 13 genes known to change expression in response to androgen in prostate cancer cells correlated between the LongSAGE and qRT-PCR data. *ADAMTS1, CENPN, CREB3L4, FKBP5, KLK3, LRIG1, NCAPD3, PAK1IP1*, and *RHOU *all increased levels of expression in response to androgen while *CXCR7, NTS, PRKACB*, and *ST7 *decreased in response to androgen (Figure 3B).

### Known or potential AREs in the regulatory regions of androgen-regulated genes

AR directly regulates transcription in response to androgen by binding to AREs in the promoter and/or enhancer regions of target genes. ChIP-chip database mining for suggested AREs combined with a literature search for known AREs revealed some of the genes that alter expression in response to androgen do contain AREs (Table 9). For the 87 genes identified using the cut-off p-value of 0.001 and 2-fold change in response to androgen, there were eight genes with AREs in their promoter, enhancer or intron regions[16,42,45-49]. AREs were detected in the proximity of seven genes by data mining of ChIP-chip studies of ARE on chromosomes 19, 20, 21, 22 [50,51]. Additionally, sequence analysis of the promoters [52] found eight genes from our gene list to contain potential AREs (Table 9). Identification of potential AREs in the regulatory regions of the newly identified genes that alter expression in response to androgen (*BLVRB, C19orf48, HM13, SOD1*) may be directly regulated by AR.

**Table 9:** Genes with confirmed or potential AREs that change expression in response to androgen

**HGNC Gene Symbol**	**Access. No.**	**Expression change by R1881 stimulation**	**Chromosome**	Distance of the ARE from TSS* (bp)	**Reference**
Conventional					

*B2M*	NM_004048	2.3	15	-1902	[42]
*NKX3-1*	NM_006167	20	8	-3013	[42]
*KLK3*	NM_001648	7.3	19	-170	[16,48]
				-4006	
				-4075	
				-4115	
*FKBP5*	NM_004117	20	6	65.6 k (Intron 5)	[47]
*NDRG1*	NM_006096	70	8	-984	[45]
*TMEPAI*	NM_020182	20	20	-2134	[45]
*TMPRSS*2	NM_005656	10	21	-148	[46]
*TPD52*	NM_005079	4	8	-359	[49]

Identified by ChIP-Chip					

*BLVRB*	NM_000713	2.1	19	-56.7 k	[51]
*C19orf48*	NM_199249	3.1	19	-363 k	[51]
*CAPNS1*	NM_001003962	2.5	19	-165 k	[51]
*HM13*	NM_178580	20	20	-330 k	[51]
*ADAMTS*1	NM_006988	4.2	21	276 k	[50]
				310 k	
				481 k	
*TMPRSS*2	NM_005656	10	21	-1063 k	[50]
				-462 k	
				13.5 k	
*SOD1*	NM_000454	-2.1	21	-496 k	[50]

Potential based on the sequence					

*B2M*	NM_004048	2.3	15	-440	[52]
*NDRG1*	NM_006096	70	8	-1018	[52]
*NKX3-1*	NM_006167	20	8	-1272	[52]
*SORD*	NM_003104	5.1	15	-1995	[52]
*TMEPA*I	NM_020182	20	20	-225	[52]
*TMPRSS*2	NM_005656	10	21	-771	[52]
*TPD52*	NM_005079	4	8	-609	[52]
*TSC22D*1	NM_006022	6	13	-1711	[52]

*TSS: Transcription starting site

### Cell-type specificity of gene expression

**Figure 4:**
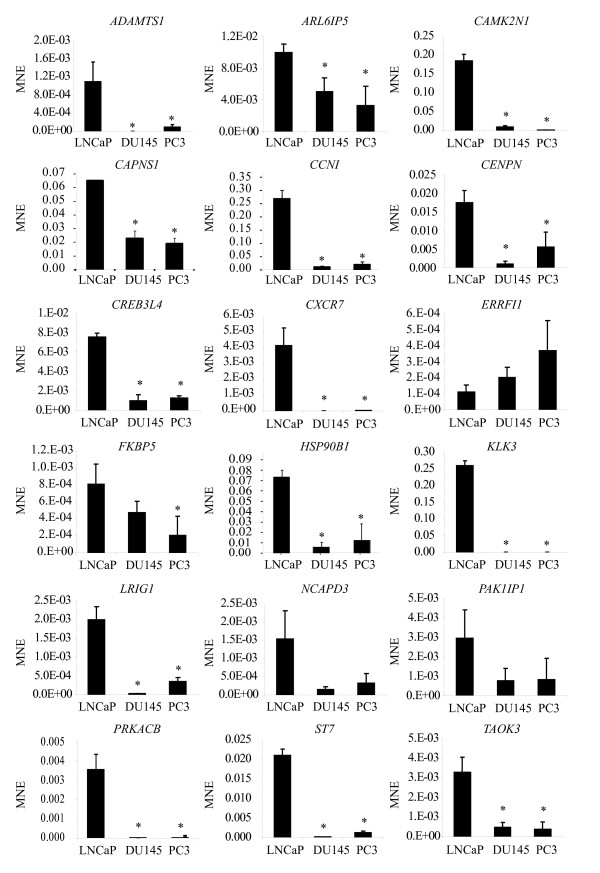
**Differential expression of candidate genes in LNCaP, DU145, and PC-3 cells**. Levels of transcripts in LNCaP, DU145, and PC-3 cells were analyzed by qRT-PCR. Error bars represent ± standard deviation(SD) for biological triplicates. [*] Asterisks indicate the significant differential gene expression in each cell line compared to LNCaP cells according to the Two-Sample Student's T-test (p ≤ 0.05) for equal (unpaired) or unequal variance as determined appropriate with the F-test.

To determine if expression of candidate genes was unique to LNCaP cells, we assayed for constitutive levels of expression of 18 known and novel candidate genes in prostate cancer cell lines DU145[53] and PC-3[54] using qRT-PCR (Figure 4). Genes chosen included those that both increased (*ADAMTS1, CAPNS1, CENPN, CREB3L4, ERRFI1, FKBP5, HSP90B1, KLK3, LRIG1, NCAPD3, PAK1IP1*, and *TAOK3*) and decreased expression in response to androgen (*ARL6IP5, CAMK2N1, CCNI, CXCR7, PRKACB *and *ST7*). No obvious trends were observed depending on whether expression of the genes increased, or decreased, in response to androgen. All genes tested, except *ERRFI1*, were expressed at a lower level in PC-3 and DU145 cells relative to LNCaP cells. This suggests that the majority of genes that alter levels of expression in response to androgen were enriched in LNCaP cells relative to PC-3 and DU145 cells. These data are consistent with both DU145 and PC3 cells being androgen-insensitive and lacking a functional AR[53,54].

### *In vivo *changes in gene expression in response to androgen-deprivation

**Figure 5:**
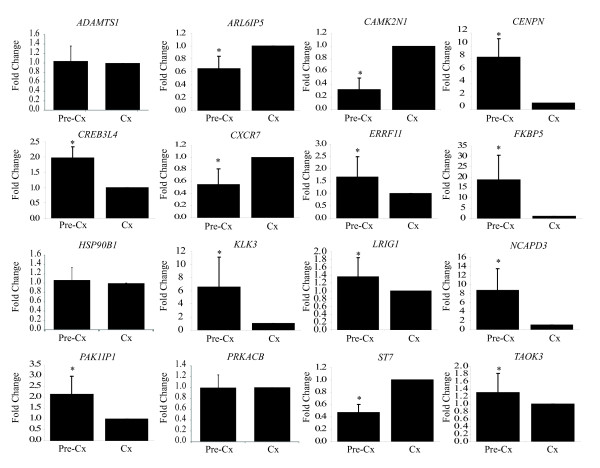
**Androgen regulation of genes in the *in vivo *Hollow Fibre model of prostate cancer**. Levels of transcripts in LNCaP cells from the Hollow Fibre model were analyzed by qRT-PCR. Cx, castrated mice, 10 days post castration, n = 12; Pre-Cx, pre-castration, day 0 of castration, n = 15. Exception: *LRIG1 *gene expression in Cx samples was represented by 11 mice. Fold-change was calculated by normalizing the mean normalized expression (MNE) of transcripts in the Pre-Cx sample to the castrate sample. In doing this, the Cx sample fold-change became one and standard deviation (SD) zero. Error bars represent ± SD. [*] Asterisks indicate the significant differential gene expression with respect to Cx according to the Two-Sample Student's T-test (p ≤ 0.05) for unequal variance.

The LNCaP Hollow Fibre model combined with qRT-PCR was employed to capture *in vivo *gene expression representative of physiological levels and castrated levels of androgen (Figure 5). We expected that the genes that had increased levels of expression *in vitro *in response to androgens, would decrease expression *in vivo *in response to castration (androgen-deprivation). Conversely, we expected that the genes that had decreased levels of expression *in vitro *in response to androgens, would increase expression *in vivo *in response to castration. These *in vivo *results would be consistent with androgen-responsiveness of the candidate genes. Of the candidate genes examined, 13 of 16 genes showed significant changes in gene expression in response to androgen-deprivation (Figure 5). As anticipated, expression of *ARL6IP5, CAMK2N1, CXCR7*, and *ST7 *increased, while *CENPN, CREB3L4, ERRFI1, FKBP5, KLK3, LRIG1, NCAPD3, PAK1IP1*, and *TAOK3 *decreased levels of expression in response to castration. No significant changes in gene expression *in vivo *was measured for *ADAMTS1, HSP90B1, or PRKACB*, suggesting that *in vivo*, other factors may influence their expression. Alternatively, the expression kinetics of each specific gene and half-life of its transcript may vary considerably. The time of harvesting samples and measuring changes in expression of genes in response to androgen-deprivation was at 10 days *in vivo *compared to 16 hr *in vitro *in response to addition of androgens (10 nM R1881). Different levels of androgen may also have profound effects on proliferation and differentiation. Physiological levels of androgen in male Nude mice may be considerably lower than the levels used *in vitro*. Androgen at 10 nM inhibits proliferation of LNCaP cells *in vitro *while 0.1 nM is optimal for proliferation[55].

## Conclusion

Androgens are essential for the growth, development and maintenance of the prostate. Here, we created LongSAGE libraries to obtain quantitative gene expression profiles of LNCaP human prostate cancer cells treated with, or without, androgen and revealed the following: 1) 33,385 tag types in the R1881 LongSAGE library and 31,764 tag types in the vehicle LongSAGE library; 2) the majority (64% to 67%) of tag types in each LongSAGE library were singletons which may represent very low abundance transcripts (≤ 5 transcript copies per cell); 3); when mapped tags were clustered and ambiguous mappings were removed, the tag types in the R1881 and vehicle LongSAGE libraries represented 7,484 genes and 7,441 genes, respectively; 4) 53% of tags mapped solely to known exons, 9% solely to known introns (novel transcript variants), and 38% to intergenic regions (novel genes or transcript variants); 5) the most highly abundant LongSAGE tag mapped to human mitochondrial NADH ubiquinone oxidoreductase chain 4 involved in metabolic energy; 6) the lower abundance classes were enriched for genes with functions in regulating transcription; 7) 87 genes were differentially expressed by two-fold (p ≤ 0.001) in response to androgen representing 0.34% of the total tag types (131 differentially expressed tag types/38,574 total tag types); 8) some of these genes have confirmed or potential AREs; 9) novel androgen regulated genes (direct or indirect) identified and validated were *ARL6IP5, BLVRB, C19orf48, C1orf122, C6orf66, CAMK2N1, CCNI, DERA, ERRFI1, GLUL, GOLPH3, HM13, HSP90B1, MANEA, NANS, NIPSNAP3A, SLC41A1, SOD1, SVIP, TAOK3, TCP1, TMEM66, USP33*, and *VTA1*; 9) expression of *ADAMTS1, ARL6IP5, CAMK2N1, CAPNS1, CENPN, CREB3L4, CCNI, CXCR7, FKBP5, HSP90B1, KLK3, LRIG1, NCAPD3, PAK1IP1, PRKACB, ST7*, and *TAOK3 *was increased in LNCaP cells compared to prostate cancer cells lacking a functional AR; and 10) significant differences in levels of expression of *ARL6IP5, CAMK2N1, CENPN, CREB3L4, CXCR7, ERRFI1, FKBP5, KLK3, LRIG1, NCAPD3, PAK1IP1, ST7*, and *TAOK3 *were measured *in vivo *in response to androgen-deprivation. The products of these genes are involved in amino acid and protein synthesis, cofactor transport, protein trafficking, response to oxidative stress, as well as signaling pathways that regulate gene expression, proliferation, apoptosis, and differentiation. These genes are potentially critical for the function and maintenance of the prostate and represent targets for clinical intervention.

## Methods

### Cell culture

LNCaP human prostate cancer cells (American Type Culture Collection, Bethesda, MD, USA) were maintained in RPMI-1640 media (Stem Cell Technologies, Vancouver, BC, Canada) supplemented with 10% v/v fetal bovine serum (FBS; HyClone, Logan, UT, USA), 100 units/mL penicillin and 100 units/mL streptomycin (antibiotics; Invitrogen, Burlington, ON, Canada). DU145 and PC-3 human prostate cancer cells were maintained in DMEM (Stem Cell Technologies) supplemented with 10% v/v FBS and 5% v/v FBS, respectively with antibiotics. All cells were maintained at 37°C with 5% CO_2_.

### Long serial analysis of gene expression

#### RNA sample generation

1 × 10^6 ^LNCaP cells were seeded in 10 cm-diameter dishes. The next day, cells were serum-starved (0% serum) for 48 hours and then treated for 16 hours with 10 nM synthetic androgen R1881 (also known as methyltrienolone; PerkinElmer, Woodbridge, ON, Canada), or solvent (vehicle) control, ethanol (final concentration 2.85 × 10^-4^%). Total RNA was extracted using TRIZOL Reagent (Invitrogen) following the manufacturer's instructions. RNA quality and quantity were assessed using the Agilent 2100 Bioanalyzer (Agilent Technologies, Mississauga, ON, Canada) and RNA 6000 Nano LabChip kit (Caliper Technologies, Hopkinton, MA, USA).

#### LongSAGE library production

LongSAGE[25] libraries were constructed with 5 μg of total RNA using the Invitrogen I-SAGE Long kit and protocol with alterations as previously published[35]. Briefly, double-stranded cDNA was synthesized from total RNA and digested with Nla III. The sample was split in half and linkers type I and II were added and ligated to Nla III overhangs. An Mme I digestion resulted in 17-21 base-pair (bp) LongSAGE tags. The tags with unique linkers were combined and ligated together to form ditags. Ditags (131 bp) were amplified with primers designed to recognize sequences within linkers type I and II using PCR. This scale-up PCR was performed in 48 wells of a 96 well plate (50 μL/well) using a 1/20^th ^dilution of template cDNA and 25 and 27 cycles of PCR (R1881 and vehicle LongSAGE library, respectively). Following an Nla III digestion to remove the linkers, the 36 bp ditags were concatenized. Concatemers sized 1300-1700 bp were digested with Nla III (1 minute) to increase the efficiency of cloning into pZErO-1 vectors. Cloned concatemers were transformed into One Shot TOP10 Electrocompetent *Escherichia coli *and colonies were picked with the Q-Pix robot (Genetix) and cultured in 2× Yeast-Tryptone media with 50 μg/mL zeocin and 7.5% (v/v) glycerol.

#### Sequencing

Glycerol stocks of transformed bacteria were used to inoculate larger cultures for alkaline lysis plasmid preparation[56]. Plasmid preparations were separated by agarose gel electrophoresis and visualized by ultraviolet light and sybr green. 1/24^th ^BigDye v3.1 terminator cycle sequencing reactions were performed with tetrad thermal cyclers (BioRad, Waltham, MA, USA) and visualized with capillary DNA sequencers, models 3700 and 3730 xl (Applied Biosystems, Foster City, CA, USA). Each library was sequenced to a depth of ~100,000 LongSAGE tags. Flanking vector sequences were removed and the LongSAGE tags were extracted from each sequence read. On average, 34 and 38 LongSAGE tags were sequenced in each read (R1881 and vehicle libraries, respectively). Sequence data were filtered for non-recombinant clones.

#### Gene expression analysis

LongSAGE expression data was analyzed with DiscoverySpace 3.2.4 and 4.01 software . Duplicate ditags (identical copies of a ditag) and singletons (tags counted only once) were retained for analysis. Sequence data were filtered for bad tags (tags with one N-base call) and linker-derived tags (artifact tags). Only LongSAGE tags with a sequence quality factor (QF) greater than 95% were included in analysis[35]. Where indicated, a clustering algorithm was used to amalgamate 1-off tags (tags one bp incorrect from a complete map to a transcript) with likely 'parent' tags to improve the mapping capability of LongSAGE tags by apparently reducing PCR/sequencing errors[35]. This clustering algorithm altered the number of tag types (i.e., species) without changing the total number of tags. In instances where clustering was used, the 95% QF cutoff was not. To filter data for candidate transcript validation, a p-value cutoff (p ≤ 0.001) was employed according to the Audic and Claverie test statistic[40]. The Audic and Claverie statistical method was used to identify differentially expressed tags between LongSAGE libraries because the method takes into account the sizes of the libraries and tag counts. LongSAGE tags that mapped ambiguously to more than one gene, and tags that differed by less than 2-fold were excluded from the candidate list. LongSAGE tags were mapped to reference sequence (RefSeq; May 30^th^, 2005) and Ensembl Gene (v31.35d), unless otherwise stated.

### Quantitative real-time polymerase chain reaction

qRT-PCR was performed on TRIZOL-extracted RNA from LNCaP (serum-starved ± R1881 or the exception in Figure 4 in 10% serum), DU145 (10% serum) and PC-3 (5% serum) cells maintained *in vitro*, and LNCaP cells maintained in the *in vivo *Hollow Fibre model[29] (see below). Contaminating genomic DNA was removed from *in vitro *RNA samples using DNA-free or TURBO DNA-free (Ambion, Austin, TX, USA). Input RNA (1 μg) was reverse transcribed with SuperScript III First Strand Synthesis kit (Invitrogen). A 10 μL qRT-PCR reaction included 1 μl of template cDNA (0.1 μL for limited LNCaP Hollow Fibre samples), 1× Platinum SYBR Green qPCR SuperMix-UDG with ROX (Invitrogen) and 0.3 μM each of forward and reverse intron-spanning primers that produce products between 85-115 bp in size (see Additional file 1 for primer sequences). qRT-PCR reactions were cycled as follows in a 7900 HT Sequence Detection System (Applied Biosystems): 50°C for 2 min, 95°C for 2 min, (95°C for 0.5 min, 55-56°C for 0.3-0.5 min, and 72°C for 0.5 min) for 40-45 cycles, 95°C for 0.25 min, 60°C for 0.25 min, and 95°C for 0.25 min. All qRT-PCR reactions were performed in technical triplicates for each of at least three biological replicates. cDNAs (from different conditions) and genes [target and reference (glyceraldehyde-3-phosphate, *GAPDH*)] to be directly compared were assayed in the same instrument run. No-template reactions (negative controls) were run for each gene to ensure that DNA had not contaminated the qRT-PCR reactions. Only qRT-PCR data with single-peak dissociation curves were included in this analysis. Efficiency checks were performed for each primer pair in each cell line. PCR products were sequenced to verify the identity of quantified transcripts. The two-tailed, two-sample Student's T-tests were performed to identify significant differences in transcript expression. The F-test was used to identify unequal variance among samples to be compared.

### LNCaP Hollow Fibre model

#### Animals

Five-week-old male athymic BALB/c Nude mice were obtained from Taconic Farms (Hudson, NY, United States of America) and kept in the British Columbia Cancer Research Centre (Vancouver, BC, Canada). Mice were maintained on a Harlan/Teklad irradiated diet with a constant supply of autoclaved water and housed in cages (three animals/cage) at 21°C ± 3°C with light/dark cycling (light between 6 AM and 6 PM). All animal experiments were performed according to a protocol approved by the Committee on Animal Care of the University of British Columbia.

#### Hollow fibre model

Polyvinylidene difluoride hollow fibres (*M*_r _500,000 molecular weight cutoff; 1-mm internal diameter; Spectrum Laboratories, Rancho Dominguez, CA, USA) were prepared and implanted as previously described[29]. Briefly, LNCaP human prostate cancer cells (3 × 10^7 ^cells) at passage 47 (provided by Dr. L.W.K. Chung at the Emory University School of Medicine, Atlanta, GA, USA) were injected into hollow fibres. The fibres were sealed and subcutaneously (s.c.) implanted into mice. Seven days post fibre implantation (day zero), mice were either castrated or left intact as controls. Blood was drawn via the tail vein each week to measure serum KLK3 levels to monitor the response to castration. Serum KLK3 levels were determined by enzymatic immunoassay kit (Abbott Laboratories, Abbott Park, IL, USA). Bundles of fibres were removed at day zero (Pre-Cx; four fibres) and day 10 (Cx; four fibres). Total RNA was isolated immediately from cells harvested from the fibres. Compromised fibres that were contaminated with mouse cells, as indicated by an infiltration of red blood cells that was determined by visual inspection, were not used in this study.

## Abbreviations

AR: androgen receptor; AREs: androgen response elements; ARL6IP5: ADP-ribosylation like factor-6 interacting protein 5; CAMK2N1: calcium/calmodulin-dependent protein kinase II inhibitor 1; CI: confidence interval; Cx: castration; ERRF11: ERBB receptor feedback inhibitor; FBS: fetal bovine serum; FDR: false discovery rate; GAPDH: glyceraldehyde-3-phosphate; GLUL: glutamate-ammonia ligase; GOLPH3: golgi phosphoprotein 3; HM13: Histocompatibility (minor) 13; HSP90B1: heat shock protein 90 kDa beta member 1; KLK3: kallikrein 3 = PSA; LDT: linker-derived tag; LongSAGE: long serial analysis of gene expression; MANEA: mannosidase: endo alpha; MNE: mean normalized expression; NANS: n-acetylneuraminic acid synthase; NIPSNAP3A: nipsnap homologue 3A; PCR: polymerase-chain reaction; PSA: prostate-specific antigen = KLK3; QF: quality factor; qRT-PCR: quantitative real-time polymerase chain reaction; R1881: methyltrienolone: synthetic androgen; RefSeq: reference sequence; SAGE: serial analysis of gene expression; SD: standard deviation; shortSAGE: short serial analysis of gene expression; SLC41A1: solute carrier family 41: member 1; SOD1: superoxide dismutase 1; SVIP: small VCP/p97-interacting protein; TAOK3: tao kinase 3; TCP1: T-complex 1; VTA1: vps20-associated 1.

## Authors' contributions

TLR conducted the experiments, analyzed the data and wrote the manuscript. GW generated the total RNA, analyzed the data, and helped to draft the manuscript. MAM provided support for the SAGE library construction with sequencing by RAH. SJM aided in the analysis of data. MDS conceived the study, designed the experiments, and coordination and wrote the manuscript. All authors read and approved the final manuscript.

## Author's Information

Tammy L. Romanuik, PhD: Graduate Student, Genome Sciences Centre, BC Cancer Agency

Current address: Medical Genetics, UBC

Gang Wang, PhD: Postdoctoral fellow, Genome Sciences Centre, BC Cancer Agency

Robert A. Holt, PhD: Head of Sequencing, Genome Sciences Centre, BC Cancer Agency

Steven J. M. Jones, PhD: Associate Director/Head of Bioinformatics, Genome Sciences Centre, BC Cancer Agency

Marco A. Marra, PhD: Director, Genome Sciences Centre

Marianne D. Sadar, PhD: Program Leader for Prostate Cancer Research, BC Cancer Agency

## Supplementary Material

Additional file 1 **Primer sequences and amplification product sizes for candidate transcripts**. The data provided represent the primer sequences used in quantitative real-time polymerase chain reaction to validate changes in gene expression in response to androgen. Click here for file
