# Supplementary material for: Identification of novel androgen-responsive genes by sequencing of LongSAGE libraries
Source: BMC Genomics. 2009 Oct 15;10:476. doi: 10.1186/1471-2164-10-476 (PMC2766392; doi:10.1186/1471-2164-10-476)
Supplement: Additional file 1 — Primer sequences and amplification product sizes for candidate transcripts. The data provided represent the primer sequences used in quantitative real-time polymerase chain reaction to validate changes in gene expression in response to androgen. [file 1471-2164-10-476-S1.PDF]

Additional File 1 Primer sequences and amplification product sizes for candidate transcripts

Gene	RefSeq Access. No.	Forward Primer (5'-3')	Reverse Primer (5'-3')	Product Size	Exons [ǀ]
ADAMTS1	NM_006988	ACTGCAAGGCGTAGGACAG	CCACAAGCATGGTTTCCAC	92	1-2
ARL6IP5	NM_006407	CATGTTTGGAGGAGTCATGG	GAGGTTCCGAAGTCTCAACG	91	2-3
BLVRB	NM_000713	GAAGTACGTGGCTGTGATGC	CCAGGTCATGTTTGGAGATG	113	4-5
C1orf122	NM_198446	AGCTCCTGGACACCATCG	GCTCCAGGTTTGGCTGAGAC	103	2-3
C19orf48	NM_199249	AAGGGCCTGACCATCACTC	ACGCCTAGGCAGGAAACAG	96	1-2
C6orf66	NM_014165	AAAGATGAAAAGCTGCTGTCG	CTGAATTCCTTCGGCTCTTG	113	2-3
CAMK2N1	NM_018584	TGCAGGACACCAACAACTTC	GCACGTCATCAATCCTATCATC	114	1-2
CAPNS1	NM_001003962	AGATGGCACTGGACAAATCC	TCCTATAGCAAGGCAGTGAGG	106	10-11
CCNI	NM_006835	TCATTCCTGATTGGCTTTCTC	GAAAGGTGATGTGCCACAAG	103	6-7
CENPN	NM_018455	ATACACCGCTTCTGGGTCAG	TGCAAGCTTTCTTCATTTCG	99	6-7
CREB3L4	NM_130898	TTCCAGAGTCGACCAGAAGC	TGTTACGTCCTTGTGGGTCA	87	9-10
CXCR7	NM_020311	CCCGGAGGTCATTTGATTG	GCTGATGTCCGAGAAGTTCC	87	1-2
DERA	NM_015954	AGTGGCTGAAGCCAGAACTC	AAGCTGCATATCTTCCAGTCAC	99	8-9
ERRF11	NM_018948	CCGATAACCATGGCCTACAG	ATTCATCGGAGAGATTTGG	87	3-4
FKBP5	NM_004117	CGCAGGATATACGCCAACAT	GAAGTCTTCTTGCCCATTGC	86	11-12
GAPDH	NM_002046	CTGACTTCAACAGCGACACC	TGCTGTAGCCAAATTCGTTG	114	8-9
GLUL	NM_002065	TGCCATACCAACTTCAGCAC	TGCCGCTTGCTTAGTTTCTC	89	6-7
GOLPH3	NM_022130	CTCCAGAAACGGTCCAGAAC	CCACCAGGTTTTAGCTAATCG	114	3-4
HM13	NM_178580	GGCCAAGGGAGAAGTGACAG	ATGCCTCTGTTCCCTCTTTG	95	10-11
HSP90B1	NM_003299	GCATCTGATTACCTTGAATTGG	TGGGCTCCTCAACAGTTTC	115	6-7
KLK3	NM_001648	CCAAGTTCATGCTGTGTGCT	CCCATGACGTGATACCTTGA	111	4-5
LRIG1	NM_015541	GACGGCTGTGAAGAAAAAGC	CTGTGGAGTCCGGGTGATAC	92	18-19
MANEA	NM_024641	TAGCAATCGAGATGATCAAAAC	AAGAGCATTGCCAGTCTTCG	109	4-5
NANS	NM_018946	CGGTCAGTGCGTCTTGTG	ATTTTCACTTTGGCCACCAC	113	5-6
NCAPD3	NM_015261	GGGCGCTTCTTACTCTCCTC	GGGTGAGAATTTTTCTTTCTTGG	98	16-17
NIPSNAP3A	NM_015469	CCATGAGGATCCCAGAGTTG	TCAGTGGTGAAAACGATGTAGG	101	5-6
NTS	NM_006183	CCACAAAATCTGTCACAGCAG	CCTTTCCATTTTTGTCATTTCC	89	3-4
PAK1IP1	NM_017906	CGTGTCTTGGAGTGTGGCTA	AGGCTCCTTTTTGCCAATTT	113	9-10
PRKACB	NM_182948	GCCACGACAGATTGGATTG	AATTGCTGGTATCTCCAGAGC	89	9-10
PRNPIP	NM_024066	CCTCAGCCTGCAACACATAG	AAGCCTCGATAGGCGAGTG	92	6-7
RHOU	NM_021205	CCCGTGAGACTCCAACTCTG	TGAAGCAGAGCAGGAAGATG	100	2-3
SLC41A1	NM_173854	GCACACCACCCTCACACTC	TCCAGTCTGCGATGTACAGG	89	10-11
SOD1	NM_000454	CCCAGGTTAACCCAGAACG	ACCCCTGCTTGTTTGTTGTC	88	4-5
ST7	NM_018412	CGGAACTTATGGGGGTCTTC	ACAGACTGGATGGGAGGATG	102	14-15
SVIP	NM_148893	AGGGTTCTCAAGCTGTCGTC	TGCAAGCTTTGCTCTTTTCTC	101	1-2
TAOK3	NM_016281	CGCAGAGCACACCTTGAG	CGCTCTTGCCTTTCCAATAG	98	20-21
TCP1	NM_030752	TGTGGCCGATGTGTCTATTG	ACCTTTGCCCAAGTCATCTG	109	11-12
TMEM66	NM_016127	GGGCAGCTATTCGGTATGTTC	TGCATCCAGTGTTTGACTCC	110	5-6
USP33	NM_201624	AAATGTGGTAATGTGATGCTTAGG	GGTCGCAGGATAACTTCAGG	113	23-24
VTA1	NM_016485	CGCACTTTTCAATACAATTTCC	CATCTTCATACTGCAAAGCACTG	110	10-11

ǀ Exons according to Ensembl
